# Simultaneous shaping of amplitude and phase of light in the entire output plane with a phase-only hologram

**DOI:** 10.1038/srep15426

**Published:** 2015-10-21

**Authors:** Liang Wu, Shubo Cheng, Shaohua Tao

**Affiliations:** 1 School of Physics and Electronics, Central South University, Changsha 410083, China; 2 Hunan Key Laboratory of Super Microstructure and Ultrafast Process, Central South University, Changsha 410083, China

## Abstract

An iterative beam shaping algorithm is proposed to simultaneously shape the amplitude and phase of an optical beam. The proposed algorithm consists of one input plane and two completely overlapped output planes which refer to the output plane in real space. The two output planes are imposed with both amplitude and phase constraints, and the constrained areas in the two output planes are complementary. As a result, both the amplitude and phase in the entire output plane are controllable and arbitrary target complex amplitudes can be achieved with the proposed algorithm. The computing result of the proposed algorithm is a phase-only distribution which can be conveniently realized with a spatial light modulator or a fabricated diffractive optical element. Both simulations and experiments have verified the high performance of the proposed algorithm.

Beam shaping techniques have promoted laser applications in various fields including materials manufacturing, laser micro-machining, and optical trapping. A fabricated diffractive optical element or spatial light modulator (SLM) can be used to modulate the wavefront of a laser beam. Designing a phase-only hologram to generate a predesigned beam in diffraction field is commonly accomplished by beam shaping algorithms. The most representative beam shaping algorithm is Gerchberg-Saxton (GS) algorithm^1^. However, the conventional GS or GS-based beam shaping algorithms can only shape the intensity or phase of a laser beam in the target diffraction field.

Beam shaping techniques play an important role in optical tweezers. Specially shaped optical beams with gradient intensity or phase can be used to trap or rotate particles^2^. Ref. 3 demonstrated that particles could be driven by force arising from phase gradient of a line or vortex beam. Optical beams designed with both intensity and phase gradients are able to achieve more versatile optical manipulation of particles^4^,^5^,^6^ than those with intensity gradient only. As phase gradient force can drive particles along intensity paths, shaping of both amplitude and phase of an optical beam is significant in optical tweezers^7^,^8^. Many techniques were proposed to shape the complex amplitude of an optical beam, Ref. 9 used two liquid-crystal devices, which make the system complicate and expensive. Although Ref. 10 achieved an excellent result with a single SLM, the optical setup required careful adjustment as light was incident to the SLM twice. In Ref. 11 researchers generated a complex hologram with Fresnel diffraction and then converted the hologram to a phase-only one for beam shaping. However, application of the method with Fraunhofer diffraction was not discussed. Researchers^12^ designed and fabricated a non-standard SLM to efficiently shape complex amplitude of a beam, but the SLM is not commercially available.

Complex amplitude shaping algorithms are commonly divided into iterative and non-iterative types. In general, non-iterative beam shaping algorithms are faster than iterative ones. But when the non-iterative beam shaping algorithms involve integrations which cannot be substituted by fast Fourier transforms, the algorithms run much slower. For example, in Ref. 13 a beam shaping algorithm was proposed to shape Gaussian beam profiles along 3D curves, but the integral calculation which contains thousands of diffraction calculations makes the algorithm time-consuming. Furthermore, non-iterative algorithms are generally used to generate Gaussian, Bessel, or vortex beams^2^,^14^,^15^, as these particular beams have analytical solutions in the hologram plane^16^,^17^. When a target beam is designed with arbitrary distribution of complex amplitude, an analytic solution will be hardly found for a phase-only hologram which generates the target beam Ref. 18 deduced an analytical solution for curves and the resulting complex hologram was approximated with a phase-only hologram by using the shape-phase algorithm^19^. However, the shape-phase algorithm^19^ can convert only one-dimensional structured holograms, e.g., holograms for line traps, in Cartesian coordinates or circular-symmetrical holograms, e.g., holograms for ring traps, in polar coordinates^20^, so the patterns generated by the method in Ref. 18 are limited to the similar structures. Although the curve beams generated in Ref. 13 have no such limitations on symmetrical structures, the resulting holograms are also complex-valued and have to be converted with a complex field encoding algorithm^21^, which is only suitable for low frequency complex field.

Unlike the non-iterative beam shaping algorithms, iterative beam shaping algorithms can generate arbitrary beam profiles in the target diffraction fields. The modified GS algorithm proposed in Ref. 22 imposes a complex amplitude constraint on the output beam in the non-zero intensity region, but the output beam in the zero-intensity region is un-controllable and the adjustable constant used in the algorithm is difficult to obtain Ref. 23 presents a method which utilizes two cascaded phase elements to separately modulate amplitude and phase of a beam, but the stringent alignment between the two phase elements is a challenging. In Ref. 24 a GS-based algorithm puts combined amplitude and phase constraints on the output beam to control complex amplitude of a beam. However, only part of the output plane is controllable and the diffraction efficiency of the hologram is not high.

In this letter we propose an improved algorithm to control complex amplitude of a beam in the entire output plane. We use two virtual planes to represent the output plane. The two virtual planes are identical and overlapped as the output plane. Both the amplitude and phase constraints are imposed on the two output planes and the constrained areas in the two output planes are complementary, so the amplitude and phase of the beam in the entire output plane are constrained. Compared with the algorithm in Ref. 24, the proposed algorithm has two substantial improvements, the extended effective area in the output plane and the increased diffraction efficiency of the phase-only hologram. The effective beam shaping area is extended from nearly half of the output plane in Ref. 24 to the entire output plane in the proposed algorithm, so the controllable optical field for the output beam is increased significantly. When a target pattern is large and covers most of the area of the output plane, the algorithm in Ref. 24 reconstructs part of the target pattern only, but the proposed algorithm can reconstruct the whole target pattern. Since the output plane is fully made use of in the proposed algorithm, the diffraction efficiency of the hologram is much higher than that in Ref. 24. The proposed algorithm can also realize a beam with arbitrary complex amplitude. The beam shaping device for the proposed algorithm is a phase-only hologram, which can be fabricated with conventional micro-fabrication technique or displayed with a commercially available phase-only SLM. Furthermore, the proposed algorithm runs fast and can be conveniently implemented in many applications such as optical binding, nanoparticles assembly, microscopic pumps, and optomechanical micro machines.

## Results

### Principle

**Figure 1:**
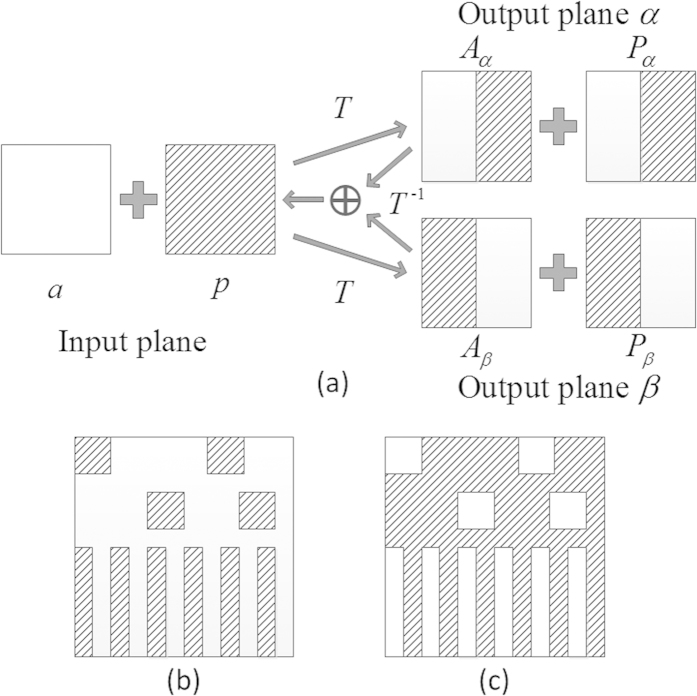
(**a**) Schematic of freedoms and constraints in the proposed algorithm. The blank regions stand for the constraint, and the slashed regions stand for the freedom. ‘*a*’ and ‘*p*’ represent domains of amplitude and phase in the input plane, respectively, ‘*A*_*α*_’ and ‘*P*_*α*_’ represent domains of amplitude and phase in the output plane *α*, respectively, and ‘*A*_*β*_’ and ‘*P*_*β*_’ represent domains of amplitude and phase in the output plane *β*, respectively. The constraints and freedoms used for (**b**) the output plane *α* and (**c**) the output plane *β*.

In Fig. 1(a) a schematic diagram is used to illustrate structure of the freedoms and constraints of a beam in the input and output planes. The blank region stands for the constraint, and the slashed region stands for the freedom. As was mentioned above, the output plane is split into two identical and overlapped planes, i.e., the output planes *α* and *β* shown in Fig. 1(a). The freedoms and constraints in the output planes *α* and *β*, shown in Fig. 1(b,c), respectively, are present as example and will be used in the algorithm. It is worth mentioning that distributions of the freedoms and constraints can be custom made.

In Fig. 1(a) the system consists of three planes: the input plane, the output plane *α*, and the output plane *β*. The output planes *α* and *β* will be merged as the output plane *αβ* in the real space. Each of the three planes consists of two domains, amplitude and phase. Domains ‘*a*’, ’*p*’, ‘*A*_*α*_’, ‘*P*_*α*_’, ‘*A*_*β*_’, and ‘*P*_*β*_’ have equal size. The domain ‘*a*’ represents constrained area of the amplitude of the input beam and the domain ‘*p*’ represents constraint-free area of the phase of the input beam. The blank regions of the domains ‘*A*_*α*_’ and ‘*P*_*α*_’ are constrained and corresponding to the half parts of the left of the amplitude and phase of the output beam, respectively, and the blank regions of the domains ‘*A*_*β*_’ and ‘*P*_*β*_’ are also constrained and corresponding to the half parts of the right of the amplitude and phase of the output beam, respectively. The constrained regions in the domains ‘*A*_*α*_’ and ‘*A*_*β*_’ are complementary, and so are the constrained regions in the domains ‘*P*_*α*_’ and ‘*P*_*β*_’. In fact, the shapes of the constrained regions in the output planes *α* and *β* can be arbitrary as long as they are complementary. For example, Fig. 1(b,c) can be used as the constraint matrixes for the output planes *α* and *β*, respectively. As the output planes *α* and *β* can be combined as the actual output plane, the amplitude and phase of the output beam in the entire output plane will be controllable.

The diffraction efficiency *η*, non-uniformity *ε*_*nu*_ of the reconstructed intensity, and relative error *ε*_*P*_ of the reconstructed phase are used to estimate performance of the beam shaping algorithm, and the definitions are shown in Eq. (1),


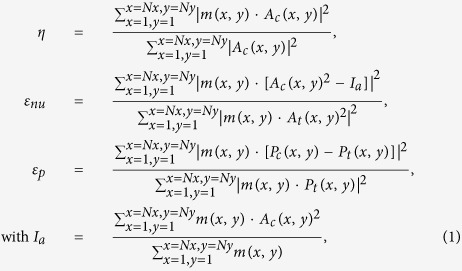


where *Nx* and *Ny* represent the total pixel numbers in *x* and *y* directions, respectively, *A*_*t*_(*x, y*) and *P*_*t*_(*x, y*) are the target amplitude and phase, respectively, *A*_*c*_(*x, y*) and *P*_*c*_(*x, y*) are the calculated amplitude and phase, respectively, and *m*(*x, y*) is a mask matrix representing the region where the target intensity is not zero. When the target intensity in pixel (*x, y*) is not zero, *m*(*x, y*) has a value of ‘1’, otherwise a value of ‘0’. *I*_*a*_ represents the average of the reconstructed intensity in the non-zero intensity region.

### Simulations

**Figure 2:**
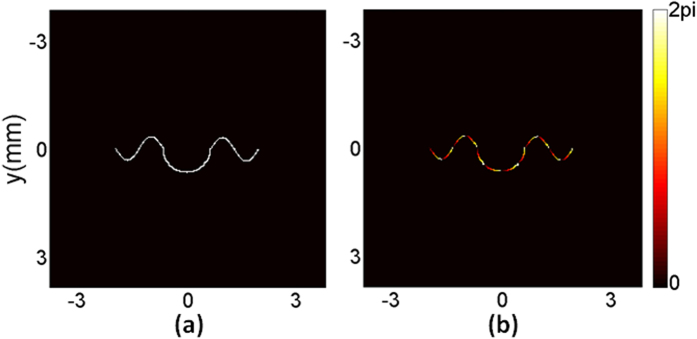
(**a**) Intensity and (**b**) phase of an arbitrary target beam.

The algorithm was coded with MATLAB 2009 in a personal computer (Intel Pentium CPU G640, 2.80 GHz). The grids of sampling points in the hologram plane and the diffraction plane were 512 × 512 pixels, and the pixel area was 15 μm × 15 μm. We used plane wave with a wavelength of 532 nm as the input beam. As shown in Fig. 2, a curve beam with uniform intensity and gradient phase is used as the target in the output plane. The beam can drive particles automatically along the curve owing to the momentum the beam possesses.

**Figure 3:**
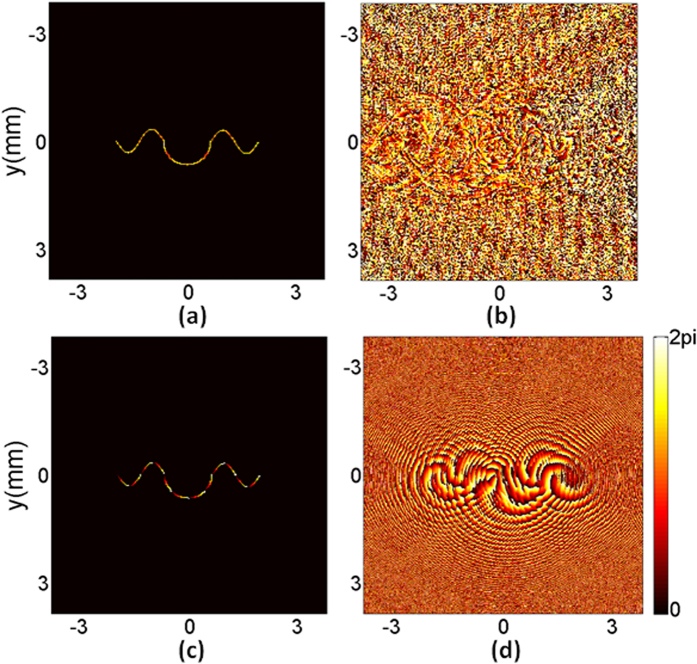
Beam shaping results of the proposed algorithm with near-field diffraction. (**a**) The reconstructed intensity, (**b**) the reconstructed phase, (**c**) the reconstructed phase in the non-zero intensity region, and (**d**) the phase-only hologram.

The proposed algorithm with near-field diffraction was used firstly to shape the designed target beam and the diffraction distance was set as 30 cm. The iteration number was 40 and the running time of the algorithm was 9.64 seconds. The simulation results are shown in Fig. 3. Figure 3(a) is the reconstructed intensity and the non-uniformity *ε*_*nu*_ is 0.64%. The diffraction efficiency *η* of the hologram is as high as 78.76%. Figure 3(b) is the reconstructed phase, which is full of noise in the area corresponding to the zero-intensity region of the target beam, but a gradient phase can still be observed in the non-zero intensity region. As phase exists only in the non-zero intensity region of a beam, the noise in the corresponding zero-intensity region in Fig. 3(b) can be ignored. We use the mask matrix *m*(*x, y*) to filter the reconstructed phase. The reconstructed phase in the non-zero intensity region is extracted and shown in Fig. 3(c). The relative error *ε*_*P*_ of the extracted phase is 3.04%. Figure 3(d) is the obtained phase-only hologram.

**Figure 4:**
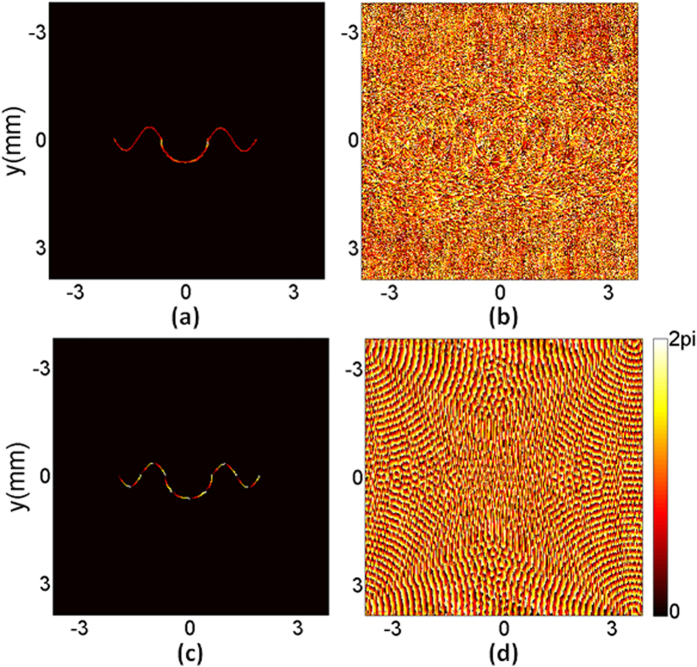
Beam shaping results of the proposed algorithm with far-field diffraction. (**a**) The reconstructed intensity, (**b**) the reconstructed phase, (**c**) the reconstructed phase in the non-zero intensity region, and (**d**) the phase-only hologram.

Then the proposed algorithm with far-field diffraction was run and the shaping results are shown in Fig. 4. The iteration number was 40 and the running time of the algorithm was 7.65 seconds. Figure 4(a) is the reconstructed intensity and the non-uniformity *ε*_*nu*_ is 3.48%. The diffraction efficiency *η* of the reconstructed intensity is still as high as 77.84%. Figure 4(b) is the reconstructed phase. Similarly, the gradient phase can still be observed in the area corresponding to the non-zero intensity region of the target beam in the output plane. The reconstructed phase in the non-zero intensity region is extracted and shown in Fig. 4(c). The relative error *ε*_*P*_ of the extracted phase is 3.77%. Figure 4(d) is the obtained phase-only hologram.

Although the target patterns presented in the simulations have mirror symmetry, the proposed algorithm has high performance for asymmetric patterns, too. Different target patterns may have a fluctuation in the quality of the reconstruction, but a target pattern with symmetry or asymmetry has nearly no impact on the reconstruction with the proposed algorithm.

For comparison, the algorithm in Ref. 24 was also used to shape the same target beam. In the near-field diffraction, the non-uniformity *ε*_*nu*_ is 0.04%, the relative error *ε*_*P*_ is 1.62%, and the diffraction efficiency *η* is only 5.60% for the algorithm^24^; in the far-field diffraction, the non-uniformity *ε*_*nu*_ is still 0.04%, the relative error *ε*_*P*_ is 1.63%, but the diffraction efficiency *η* is as low as 3.48%. The uniformity of the reconstructed intensity with the algorithm in Ref. 24 is better than that with the proposed algorithm for the algorithm^24^ shifts the unwanted information from the central area to the peripheral area effectively. As a result, in Ref. 24 the effective shaping area in the peripheral is sacrificed and the diffraction efficiency is low as much of the light is distributed in the uncontrolled area. In contrast, the proposed algorithm makes use of the entire output plane efficiently, so the diffraction efficiency is much higher.

**Figure 5:**
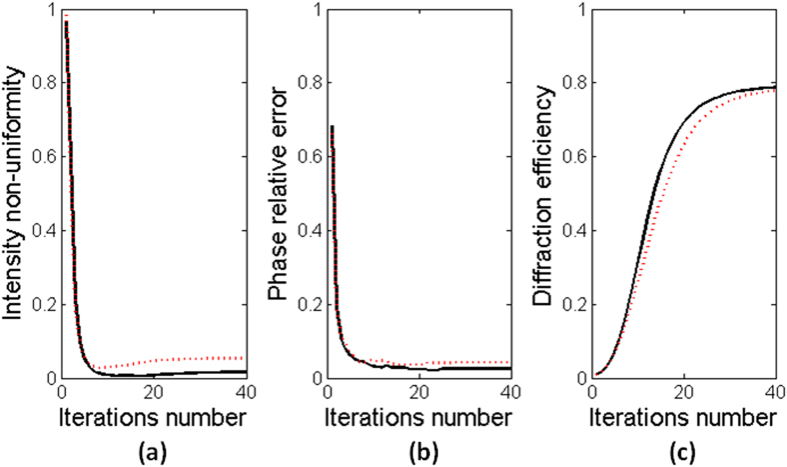
The plots for (**a**) the non-uniformity of intensity *ε*_*nu*_, (**b**) the relative error of phase *ε*_*P*_, and (**c**) the diffraction efficiency *η* versus the number of iterations. The solid lines stand for the proposed algorithm with near-field diffraction and the dotted lines stand for the proposed algorithm with far-field diffraction.

The plots for the non-uniformity of intensity *ε*_*nu*_, the relative error of phase *ε*_*P*_, and the diffraction efficiency *η* of the proposed algorithm versus the number of iterations are shown in Fig. 5. The solid lines stand for the results with near-field diffraction and the dotted lines stand for the results with far-field diffraction. The non-uniformity *ε*_*nu*_ and the relative error *ε*_*P*_ reach a minimum value after about only 10 iterations. The diffraction efficiency *η* reaches 70% after about only 20 iterations.

### Experiments

**Figure 6:**
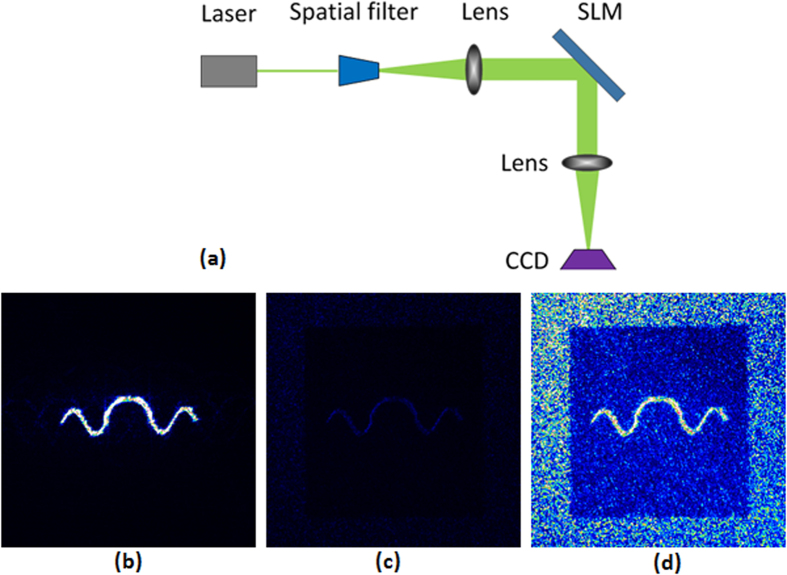
(**a**) A schematic of the experimental beam shaping setup for the far-field diffraction; (**b**) the captured intensity of the optical beam generated by the proposed algorithm when the power of laser was 1 mW; (**c**) the captured intensity of the optical beam generated by the algorithm in Ref. 24 when the power of laser was 1 mW; (**d**) the captured intensity of the optical beam generated by the algorithm in Ref. 24 when the power of laser was 22 mW.

As shown in Fig. 6(a), the beam shaping experimental setup with far-field diffraction consists of a diode-pumped laser (Coherent, Genesis MX532-1000 STM, *λ* = 532 nm), a spatial filter, two convex lenses, an SLM (Phase series from BNS), and a charge-coupled device (CCD, Newport, LBP-2-USB). The SLM has 512 × 512 pixels with a pitch of 15 μm.

Figure 6(b) is the captured intensity of the optical beam generated with the proposed algorithm when the power of laser was 1 mW. It can be seen that the curve has high intensity and is focused. Figure 6(c) is the captured intensity of the optical beam generated with the algorithm in Ref. 24 under the same condition. The captured intensity of the curve is too dim to be seen clearly because of the low diffraction efficiency. Figure 6(d) is the captured intensity of the optical beam generated by the algorithm in Ref. 24 when the power of laser was increased to 22 mW. The noise in the central areas is distinct. Although the intensity uniformity of the algorithm in Ref. 24 is better than that of the proposed algorithm in the simulations, the CCD captured intensity reconstructed with the proposed algorithm is smoother and less noisy in the central area. The size of the captured areas is about 3 mm × 3 mm.

**Figure 7:**
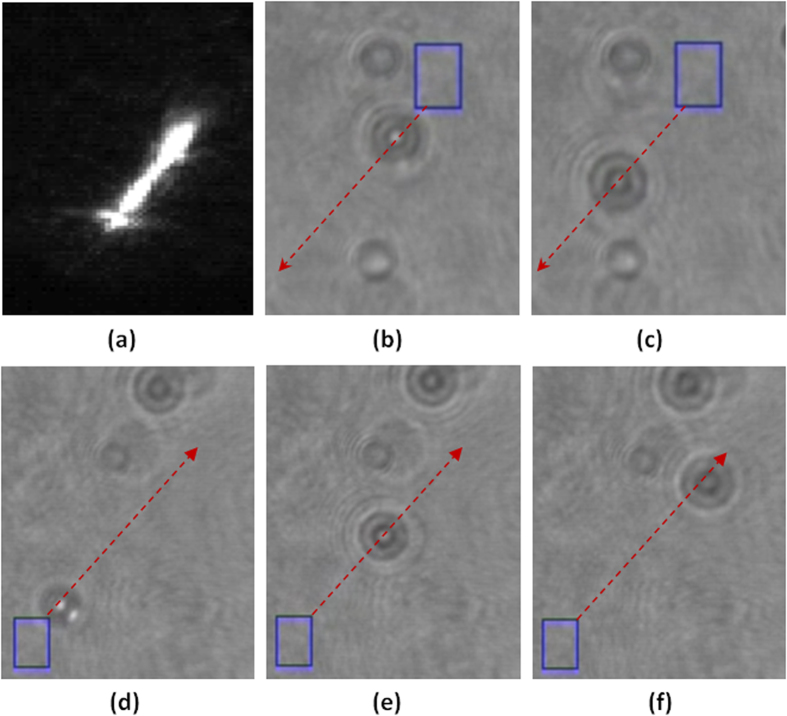
Experimental results for optical manipulation with the line beams generated by the proposed algorithm. (**a**) The CCD captured intensity of the tilted line beam. (**b**,**c**) CCD captured video frames showing that a particle moves automatically along a tilted line from the upper right to the lower left. (**d**–**f**) CCD captured video frames showing that a particle moves automatically along a tilted line from the lower left to the upper right.

Then an optical trapping experiment was used to verify the gradient phases of the reconstructed beams. The optical tweezers system mainly consists of the above mentioned experimental beam shaping system and a 100× oil-immersed objective lens (Olympus, N.A. 1.3). The optical beams used in the tweezers system were two tilted line beams with uniform intensities and gradient phases along the lines in two opposite directions and were reconstructed by the proposed algorithm with far-field diffraction. Polystyrene beads with a diameter of about 3 *μm* were immersed in deionized water of refractive index of 1.33 and used as manipulating objects. As shown in Fig. 7(a–f), two particles are trapped and driven automatically by the titled line beams from the upper right to the lower left and from the lower left to the upper right, respectively. Hence, the gradient phases of the titled line beams reconstructed by the proposed algorithm are verified. Furthermore, in our experiments special optical beams such as vortex beams and curve beams with designed intensity profiles and phase gradients have also been generated with the proposed algorithm and used to manipulate particles successfully.

## Discussion

We have proposed an iterative beam shaping algorithm which simultaneously shapes both the amplitude and phase of an optical beam in the entire output plane with a phase-only hologram. In the proposed algorithm, two completely overlapped output planes are imposed with both amplitude and phase constraints of a target beam. We implemented both the simulations and experiments to examine the performance of the proposed algorithm. In the simulation, a curve beam with uniform intensity and gradient phase was used as the target and both the near-field diffraction and far-field diffraction were used for the algorithm. The reconstructed intensities and phases by the proposed algorithm are in good agreement with the target ones. The diffraction efficiency of the proposed algorithm is as high as 78.76% in the near-field diffraction simulation and 77.84% in the far-field diffraction simulation. In the experiment, the CCD captured patterns reconstructed by the proposed algorithm are smooth, bright, and with high contrast. In the optical manipulation, two particles were trapped and transferred automatically by the tilted line beams because of the phase gradients of the beams.

## Methods

In the proposed algorithm, the calculated complex amplitude is substituted by the modified complex amplitude in the output planes *α* and *β* in the iteration. The output planes *α* and *β* are completely overlapped and can be merged as the actual output plane *αβ*. The target amplitude, calculated amplitude, target phase, and calculated phase in the output plane *αβ* are represented with *A*_*t*_, *A*_*c*_, *P*_*t*_, and *P*_*c*_, respectively. The modified complex amplitude *U*_*α*_ in the output plane *α* is expressed in Eq. (2).


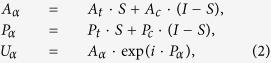


where *A*_*α*_ and *P*_*α*_ represent the modified amplitude and phase in the output plane *α*, respectively, *I* represents the unit matrix, and *S* represents the constraint matrix of the constrained areas in both the amplitude and phase domains in the output plane *α*. Matrix *S* consists of values ‘1’ and ‘0’, which represent the constrained and constraint-free pixels in the output plane *α*, respectively. The modified complex amplitude *U*_*β*_ in the output plane *β* is expressed in Eq. (3).


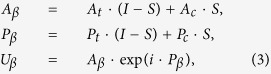


where *A*_*β*_ and *P*_*β*_ represent the modified amplitude and phase in the output plane *β*, respectively. (*I* − *S*) represents the constraint matrix of the constrained areas in both the amplitude and phase domains in the output plane *β*.

**Figure 8:**
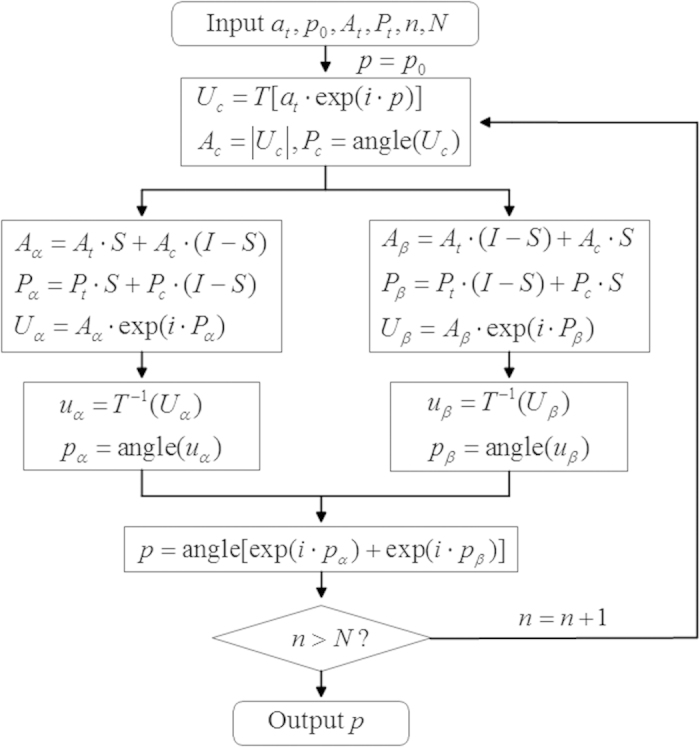
A flow chart of the proposed algorithm.

The flow chart of the proposed algorithm is shown in Fig. 8, where *n* and N are the numbers of iterations run and the total iterations, respectively. The initial phase *p*_0_ of the input beam is generated randomly. The constrained amplitude *a*_*t*_ of the input beam is usually set as uniform or a Gaussian distribution. *T* and *T*^−1^ represent the functions of the forward and backward propagations of the beam, respectively. The angular plane wave spectrum method and Fourier transforms are used to simulate the near-field and far-field diffractions, respectively. *U*_*c*_ represents the calculated complex amplitude in the output plane *αβ. u*_*α*_ and *u*_*β*_ represent the complex amplitudes in the input plane computed by the backward propagation from the modified complex amplitudes in the output planes *α* and *β*, respectively. *p*_*α*_ and *p*_*β*_ represent the phases in the input plane calculated from *u*_*α*_ and *u*_*β*_, respectively. The effective phase *p* in the input plane is obtained by Eq. (4) after each iteration run,


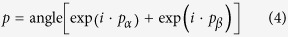


The iteration terminates when the condition *n* > *N* is satisfied and then the phase-only hologram *p* is obtained.

## Additional Information

**How to cite this article**: Wu, L. *et al.* Simultaneous shaping of amplitude and phase of light in the entire output plane with a phase-only hologram. *Sci. Rep.* **5**, 15426; doi: 10.1038/srep15426 (2015).
